# Gene-Wise Burden of Coding Variants Correlates to Noncoding Pharmacogenetic Risk Variants

**DOI:** 10.3390/ijms21093091

**Published:** 2020-04-27

**Authors:** Jihye Park, Soo Youn Lee, Su Youn Baik, Chan Hee Park, Jun Hee Yoon, Brian Y. Ryu, Ju Han Kim

**Affiliations:** Seoul National University Biomedical Informatics (SNUBI), Division of Biomedical Informatics, Seoul National University College of Medicine, Seoul 03080, Korea; mzac081225@snu.ac.kr (J.P.); soo7027@snu.ac.kr (S.Y.L.); blue880725@snu.ac.kr (S.Y.B.); chanchan@snu.ac.kr (C.H.P.); swiri021@gmail.com (J.H.Y.); brianryu87@gmail.com (B.Y.R.)

**Keywords:** drug response, genetic variability, deleterious sequence variant, pharmacogenomics, pharmacogenetics, next generation sequencing, variant burden

## Abstract

Genetic variability can modulate individual drug responses. A significant portion of pharmacogenetic variants reside in the noncoding genome yet it is unclear if the noncoding variants directly influence protein function and expression or are present on a haplotype including a functionally relevant genetic variation (synthetic association). Gene-wise variant burden (GVB) is a gene-level measure of deleteriousness, reflecting the cumulative effects of deleterious coding variants, predicted in silico. To test potential associations between noncoding and coding pharmacogenetic variants, we computed a drug-level GVB for 5099 drugs from DrugBank for 2504 genomes of the 1000 Genomes Project and evaluated the correlation between the long-known noncoding variant-drug associations in PharmGKB, with functionally relevant rare and common coding variants aggregated into GVBs. We obtained the area under the receiver operating characteristics curve (AUC) by comparing the drug-level GVB ranks against the corresponding pharmacogenetic variants-drug associations in PharmGKB. We obtained high overall AUCs (0.710 ± 0.022–0.734 ± 0.018) for six different methods (i.e., SIFT, MutationTaster, Polyphen-2 HVAR, Polyphen-2 HDIV, phyloP, and GERP^++^), and further improved the ethnicity-specific validations (0.759 ± 0.066–0.791 ± 0.078). These results suggest that a significant portion of the long-known noncoding variant-drug associations can be explained as synthetic associations with rare and common coding variants burden of the corresponding pharmacogenes.

## 1. Introduction

Person-to-person drug response variability is a major problem in pharmacotherapy, clinical trials, and drug development since it can often lead to a therapeutic failure or an adverse drug reaction (ADR). Besides common genetic polymorphisms, recent sequencing projects have revealed a plethora of rare genetic variants in genes encoding proteins. Genetic variations in genes encoding drug-metabolizing enzymes, receptors, and transporters have been associated with individual variability in drug efficacy and toxicity [1,2].

Population-based and genome-wide observational studies such as the genome-wide association studies (GWAS), are some of the most powerful tools for investigating the genetic variant-drug associations (VDAs). However, current approaches in a clinical affected vs. non-affected case-control setting are inherently limited because: (1) the numbers of genotypes, drugs, and their associations are too numerous to be reliably tested. (2) rare variants are not likely observed, which are crucial to understanding the inter-individual differences in drug response. [3] Study sizes are usually only enough to find the common variants. (3) Most of the variants identified from GWAS appear in the noncoding regions, which are not directly informative [4].

Recently, technological advances in next generation sequencing (NGS) have facilitated large-scale genetic variability studies, and pharmacological impacts of numerous genetic variants continue to be discovered. This enables alternative approaches for predicting the inter-individual drug response differences. Gene-wise variant burden (GVB), an integrated gene-level measure of the cumulative impact of the multitude of deleterious variants, including common, rare, and even novel variants on a given gene, has been successfully applied to address many pharmacogenetic problems [5,6,7,8]. Pharmacokinetic and pharmacodynamic genes associated with withdrawn, precautionary, and the US FDA-approved pharmacogenomics biomarker drugs exhibited significantly lower GVBs than those associated with safe drugs [5].

Pharmacogenomics Knowledge Base (PharmGKB) is a comprehensive manually curated catalog of variant-drug associations (VDAs) between the identified pharmacogenetic variants and the drug-related phenotypes, with varying levels of evidence from literature and ongoing research [9]. However, significant portions of the pharmacogenetic variants are common genetic polymorphisms within the noncoding genome, severely limiting their functional implications. It is unclear if the noncoding variants directly influence protein function, expression or are present on a haplotype that includes the functionally relevant genetic variations (synthetic association). Dickson et al. [10] defined *synthetic association* as the association of a genotyped common marker resulting from multiple unobserved low-frequency causal variants.

To test the potential associations between the long-known PharmGKB noncoding and the NGS-based coding variants, we computed a drug-level GVB score for each of the 5099 drugs obtained from DrugBank, by integrating the gene-level GVBs of 3668 drug-related genes with 19,038 drug-gene relations for each of the 2504 individual genomes of the 1000 Genomes Project [11]. Using the VDAs registered in PharmGKB as ‘gold standard’, we evaluated the correlations between the long-known noncoding pharmacogenetic variants (or markers) and the gene-wise burdens of newly identified rare and common coding variants aggregated into the drug-level GVBs.

We comprehensively evaluated GVB for predicting the noncoding VDAs in PharmGKB, by applying six different in silico methods (i.e., SIFT [12,13], MutationTaster [14], PolyPhen-2 HDIV [15], PolyPhen-2 HVAR [15], phyloP [16], and GERP^++^ [17]), different ethnic groups, and anatomical main groups of drugs (i.e., The Anatomical Therapeutic Chemical (ATC) Classification System). We obtained the areas under the receiver operating characteristics curves (AUCs) by comparing the drug-level GVB ranks against the long-known PharmGKB noncoding VDAs for each of the 2504 individual genomes of the 1000 Genome Project considering ethnicity or not considering ethnicity. The AUCs ranged from 0.710 ± 0.022 to 0.734 ± 0.018 for six different deleterious variant scoring algorithms. Considering ethnicity generally improved the prediction accuracies (0.759 ± 0.066 to 0.791 ± 0.078). These results suggest that a significant portion of the long-known noncoding VDAs in the PharmGKB may be explained by synthetic associations on haplotypes with rare and common coding variants. In silico drug-level GVB integrating individual genome sequences with pharmacogenetic knowledge may complement the current population-based observational pharmacogenomics research, and vice versa.

## 2. Results

**Figure 1:**
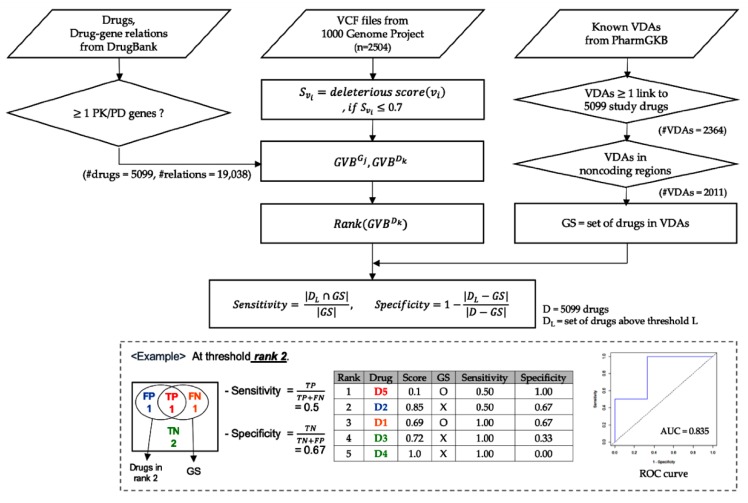
Workflow of the study. This workflow shows the steps for calculating the gene and drug GVB and evaluating scores by testing the presence or absence of the known VDAs in the PharmGKB. Abbreviations: VDA, variant-drug associations; PK, pharmacokinetics; PD, pharmacodynamics; GVB, gene-wise variant burden; AUC, area under the receiver operating characteristics curve; ROC, receiver operating characteristic; TP, true-positive (red); TN, true-negative (green); FP, false-positive (blue); FN, false-negative (orange); and GS, gold standard.

Figure 1 demonstrates the steps for computing the gene-level and the drug-level GVBs of the 3668 pharmacogenes and 5099 drugs with the 19,038 PK/PD relations obtained from the DrugBank (Figure 1, left panel), and evaluating scores by testing presence or absence of the 2011 known noncoding VDAs in the PharmGKB (right panel) for each of the 2504 individual genomes of the 1000 Genomes Project (middle panel). Figures S1 and S2 show the distributions of variant score and the GVB distributions of genes and drugs using six variant scoring algorithms.

### 2.1. GVB Score Distribution According to Pharmacogenomics Categories

**Figure 2:**
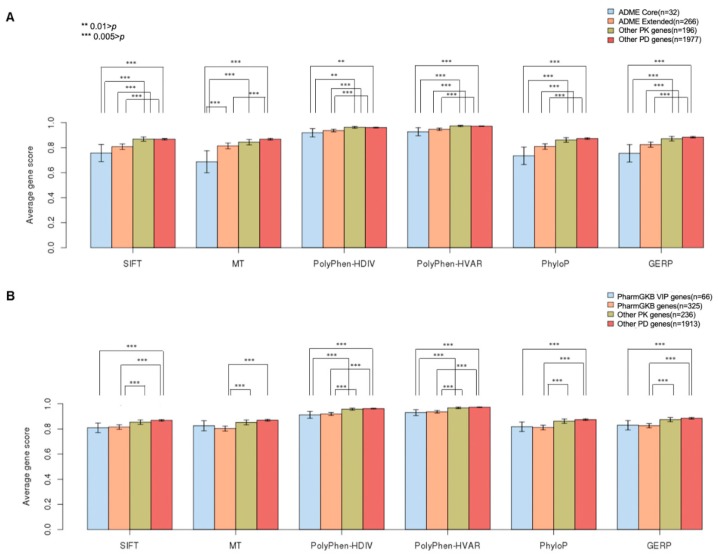
Gene-level GVB distribution in four pharmacogenetic categories across six in silico methods obtained from the 2504 individual genomes. Average GVB distributions of (**A**) ADME Core, Extended, Other PK, and Other PD genes and (**B**) PharmGKB VIP, Genes with known SNPs which affect drug efficacy from PharmGKB, Other PK, and Other PD genes across six in silico variant scoring methods (i.e., SIFT, MutationTaster, PolyPhen-2, PolyPhen-2 HVAR, phyloP, and GERP^++^). The ADME Core and the ADME Extended genes exhibited significantly lower GVBs than those in the Other PK/PD genes and the PharmGKB (VIP) genes, similarly, exhibited significantly lower GVBs than those in the Other PK/PD genes from DrugBank. *** *p* < 0.005; ** *p* < 0.01.

Pharmacogenes from DrugBank were classified as belonging either to the more strictly defined ADME Core (*n* = 32) or the ADME Extended (*n* = 266) genes [18] (Figure 2A) and the PharmGKB VIP (Very Important Pharmacogenes) (*n* = 66) or the ‘general’ PharmGKB (*n* = 325) genes, with known SNPs, which affect the drug efficacy (Figure 2B). The ADME Core and the PharmGKB VIP genes are generally regarded as having a stronger pharmacogenetic impact than that in the ADME Extended and the ‘ordinary’ PharmGKB genes, respectively. The average gene-level GVBs among the 2504 subjects, obtained using all the six in silico methods, were statistically significantly lower (*p* < 0.01) for both the ADME Core and the Extended genes than those in both the Other PK (*n* = 196) and the Other PD (*n* = 1977) genes (Figure 2A). Moreover, the ADME Core genes exhibited a tendency for lower average gene-level GVBs than the tendency for the ADME Extended genes. These findings for the ADME Core/Extended genes associated with variable drug responses are consistent with the general understanding that core pharmacogenetic genes are associated with higher inter-individual and/or inter-ethnic drug response variabilities. GVB, which applies geometric mean to individual sequences, is designed to capture the genetic variability due to a variable variant distribution in a population, by the highly weighting variants with lower (or deleterious) in silico scores, even with very low frequencies, down to singletons. Low or very low-frequency variants are frequently ignored by other gene-level approaches. Consistent with the ADME classifications, the PharmGKB VIP and the ‘general’ PharmGKB genes showed significantly lower average GVBs than those observed in the ‘ordinary’ Other PK/PD genes in DrugBank (Figure 2B). While a significant portion of the long-known VDAs in the PharmGKB has been established, considering noncoding SNPs, GVB considers only coding variants. Ab initio GVB may suggest the association between noncoding and coding variants in the PK/PD genes and their role in drug response variabilities (Figure 2).

### 2.2. Drug-Level GVB Association with the ‘Gold Standard’ Variant-Drug Associations in PharmGKB

**Figure 3:**
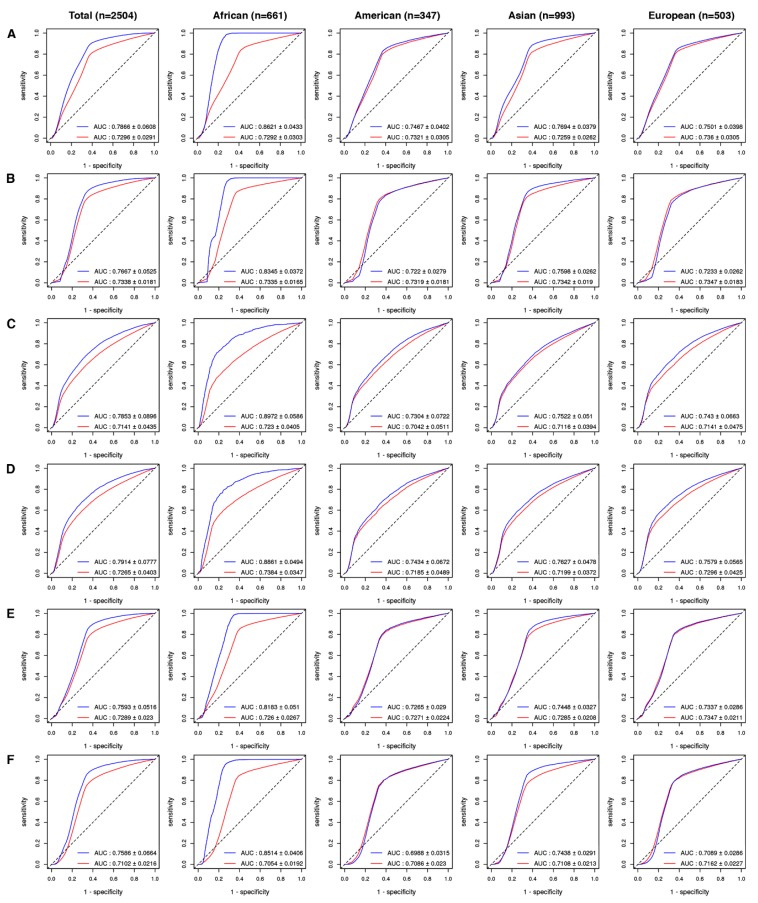
Ethnicity-specific and non-specific GVB evaluations against the variant-drug associations in PharmGKB, the gold standard, for 2504 individual genomes of the 1000 Genome Project. The AUCs (mean ± SD) were obtained from the ethnicity-specific (blue line) and the non-specific (red line) evaluations of the 5099 drugs from the DrugBank using (**A**) SIFT, (**B**) MutationTaster, (**C**) Polyphen-2 HVAR, (**D**) Polyphen-2 HDIV, (**E**) phyloP, and (**F**) GERP^++^ for all and four ethnic subgroups, including African (*n* = 661), American (*n* = 347), Asian (*n* = 993), and European (*n* = 503). The dotted line represents the reference line (AUC = 0.5). AUC: area under the receiver operating characteristics curve.

**Figure 4:**
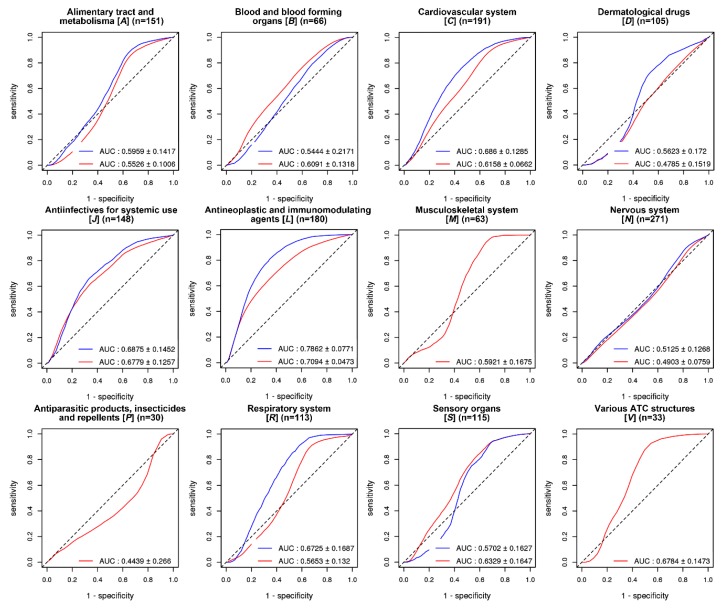
Drug-level GVB evaluation in predicting the variant-drug associations in PharmGKB across different drugs in 12 anatomical main groups. Drug-level GVB_SIFT_ for the 2504 individual genomes of the 1000 Genome Project was evaluated, according to the 14 anatomical main groups provided by ATC, two classes, [G] and [H], were excluded due to the lack of variant-drug association in PharmGKB (Table S1). The dotted line represents the reference line (AUC = 0.5). The best AUC score (0.7862) was obtained for antineoplastic and immunomodulating agents (class [L], *n* = 180). Ethnicity-specific (blue line) rather than the non-specific (red line) validations showed better AUCs, except for class [B] and [S]. AUC: area under the receiver operating characteristics curve.

**Figure 5:**
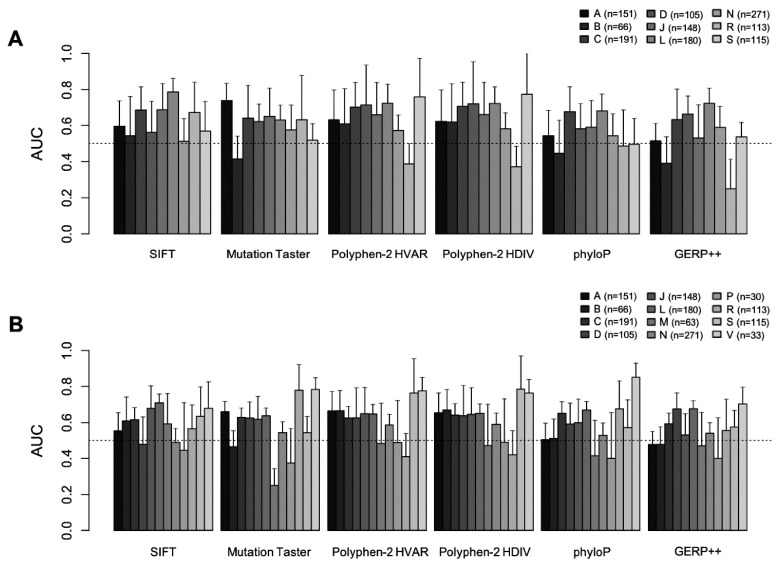
Comparison of the drug-level GVB prediction accuracies for variant-drug associations in PharmGKB across different anatomical main groups of drugs, using six different in silico methods. The AUCs for the (**A**) ethnicity-specific and (**B**) ethnicity-non-specific evaluations across the 14 anatomical main groups using six different in silico methods (i.e., SIFT, MutationTaster, PolyPhen-2 HVAR, PolyPhen-2 HDIV, phyloP, and GERP^++^). The dotted line represents the reference line (AUC = 0.5). GVB_SIFT_ exhibited robust performance across all the ATC classes, especially in the ethnicity-specific validations (all AUCs > 0.5 and AUC = 0.7862 for antineoplastic and immunomodulating agents (class [L], *n* = 180), respectively). Classes G and H were excluded due to the lack of variant-drug association (VDA) in PharmGKB and classes M, P, and V (A) due to the lack of ethnicity-specific VDA information in PharmGKB. AUC: area under the receiver operating characteristics curve.

For each of the 2504 subjects of the 1000 Genome Project, we ranked the drug-level GVBs for the 5099 drugs from DrugBank. By matching the list of VDAs from the PharmGKB [9] to each of the 2504 individual genome sequences, we created a PharmGKB drug list as gold standard with their drug-level GVBs for the 5099 drugs. We obtained the prediction accuracy profiles, including sensitivity, specificity, and the area under the receiver operating characteristic curves (AUCs, Figure 3, Figure 4 and Figure 5), by systematically comparing the GVB ranks against the gold standard PharmGKB drug list from the known pharmacogenetic VDAs (Figure 1).

AUCs obtained for all of the 5099 drugs using GVB_SIFT_ (Figure 3A) were 0.7866 ± 0.0608 and 0.7296 ± 0.0291 for ethnicity-specific (blue line) and ethnicity-non-specific (red line) validations, respectively. The ethnicity-specific validations demonstrated consistently higher AUCs than those in the non-specific ones for all of the four major ethnic subgroups, i.e., African, American, Asian, and European, of the 1000 Genomes Project. Furthermore, the GVB prediction accuracies, using five other in silico methods (Figure 3B MutationTaster, Figure 3C Polyphen-2 HVAR, Figure 3D Polyphen-2 HDIV, Figure 3E phyloP, and Figure 3F GERP^++^), showed highly consistent validation results (AUCs: 0.7586 ± 0.0664-0.7914 ± 0.0777 and 0.7102 ± 0.0216–0.7338 ± 0.0181) for ethnicity-specific and non-specific validations, respectively. The African population benefited the most from the ethnicity-specific validation, with the resulting AUC score ranging from 0.8183 ± 0.051 to 0.8972 ± 0.0586 for the six in silico methods. Please note that some GWAS genotype-phenotype associations may be validated in one specific ethnic group but not in others [19,20,21] due to ethnic differences in the linkage disequilibrium patterns with causal variants [20].

### 2.3. Evaluation of GVB Prediction Accuracies for Drugs in Different Drug Categories

The ATC classification system provides 14 anatomical main groups into which 1336 of the 5099 drugs are classified (Table S1). The overall AUC was better for the ethnicity-specific (blue line) than that in the non-specific validations (red line), except for the class [B] Blood and the blood-forming organs and [S] Sensory organs (Figure 4). The best AUC score (0.7862) was obtained for antineoplastic and immunomodulating agents (class [L], *n* = 180). Antineoplastic drugs are known to be associated with a large inter-individual toxicity and response profile variabilities, and genetic heterogeneity is one of the main contributors to these variabilities [22,23,24].

Figure 5 summarizes the AUC distributions by the different ATC anatomical main groups, using six different in silico methods. GVB_SIFT_ exhibited robust performance across all the ATC drug classes, especially in the ethnicity-specific validations (all AUCs > 0.5 and AUC = 0.7862) for antineoplastic and immunomodulating agents, respectively, (class [L], *n* = 180). Classes [G] and [H] were excluded because they had no gold standard VDAs in the PharmGKB. For classes [M], [P] and [V], no ethnicity-specific VDA information was available in the PharmGKB. Evaluation results using the other five in silico methods for the ATC anatomical main drug groups have been provided in Figures S3–S7.

## 3. Discussion 

The present study demonstrated a significant correlation between the long-known noncoding VDAs (*n* = 2011), cataloged in the PharmGKB with functionally relevant rare and common coding variants, in the corresponding 3668 pharmacogenes for the 5099 drugs from the DrugBank. We excluded coding VDAs in the PharmGKB for fair evaluations. Both the gene- and the drug-level GVBs were systematically evaluated using six different in silico methods for different ethnic groups of the 2504 subjects of the 1000 Genomes Project. Our study suggests that a significant portion of the long-known noncoding variant-drug associations could be explained as synthetic associations with rare and common coding variants in the corresponding pharmacogenes.

The vast majority of GWAS findings are noncoding variants [25]. Indeed, 88% ( = 2011/2364) of the known VDAs from PharmGKB used for our evaluation study were noncoding variants. Many noncoding variants resided in regulatory regions such as promoter, 5′-untranslated regions (5′-UTR), intronic regions, 3′-untranslated regions (3′-UTR), and intergenic regions, which control gene splicing, transcription, and translation [26]. The effects of noncoding variants on drug responses can be interpreted as the potential impact of noncoding variants on functional aspects of a coding gene [4]. Non-coding variants in the vitamin K epoxide reductase complex 1 (VKORC1) gene have been known to affect gene transcription and alter dose requirements of warfarin [27], which targets the VKORC1 gene and causes inhibition on VKORC1 activities.

In contrast, both the gene- and the drug-level GVBs are based only on the coding variants. The present study demonstrated that the gene-wise integration of the coding variant burden reliably predicted the long-known noncoding VDAs (*n* = 2011) in PharmGKB. It is evident that some of the noncoding variants directly influence the protein function or expression. However, our result suggests that a significant portion of the noncoding variants may represent a type of synthetic association created by the complex unobserved linkage associations of the functionally relevant coding variants on a haplotype genome. Further studies on these direct-functional and indirect-marker associations for variable drug responses will be needed.

The current approaches in a clinical affected vs. non-affected case-control setting are prohibitively costly for correctly evaluating all yet-unknown VDAs, given the overwhelming numbers of genetic variants, drugs, and their PK/PD associations. Thus, the establishment of an in silico method can be important and beneficial to the field, which enable us to narrow down and focus on those potentially critical gene-drug combinations. Ab initio GVB may be applied to discover unknown pharmacogenes with significant inter-individual drug response variabilities [6,7,8]. Further improvements and molecular validations should be required before use GVB as a supportive tool for drug safety scoring for a given drug for an individual or population, using NGS.

The 1000 Genomes Project provides an effective platform for the systematic analysis of sequence variations on multiple ethnic groups. Recently, increasing emphasis has been placed on the inter-ethnic differences in drug response [28]. For instance, the European ancestry has a better antihypertensive response to calcium channel blockers, compared to those in the African ancestry, whereas the African ancestry has a better response to diuretics than those in the European ancestry [29]. A specific genotype may be important in determining drug effects for one population, but not for others. Two common Beta2-adrenergic receptor (BAR2) polymorphisms, Arg16 to Gly and Gln27 to Glu, are associated with altered BAR2 response in asthma and hypertension. The Arg16 to Gly allele is more frequent in the Caucasian-American population than that in the Chinese population and Gln27 to Glu allele, more in the Caucasian-Americans than those in the African-American or the Chinese populations. Such ethnic differences in BAR2 polymorphisms explain the altered response to Beta-adrenergic receptor (BAR) agonists in different ethnic groups [30]. Ethnicity, with different genomic architectures, is one of the key parameters determining person-to-person drug response variabilities. Our results demonstrated that GVB correlates better to the ethnicity-specific VDAs than to the non-specific VDAs, which implies that coding variants are more robust to inter-ethnic variabilities than indirect noncoding ones.

Our current scoring method can be further refined by constructing networks of the drug-gene interactions and carefully weighting the edges based on a better PK/PD knowledge such as Km, Kcat, and, Vmax. Although we collected these pharmacokinetic parameters from various databases such as PubChem [31], BRENDA [32], SABIO-RK [33], and MetaCyc [34], we found that less than 10% of all the DrugBank drugs had at least one of the above pharmacokinetic parameters. Future work will be required in order to use up-to-date pharmacokinetic parameters when identified. Demographic factors, including age, gender, body weight, and ethnicity, and clinical factors such as the renal and hepatic functions, should also be integrated in order to improve the accuracy of the method and its potential clinical relevance.

In summary, we suggest that a significant portion of the long-known noncoding pharmacogenetic markers are indeed ‘synthetic’ representations of the deleterious coding variant burdens on the corresponding pharmacogenes. We used GVB, which is a gene-level measurement of deleteriousness, representing the cumulative effects of genetic variants by using next generation sequencing (NGS) data. We comprehensively evaluated the validity of the method using the known pharmacogenomics VDAs in the PharmGKB, as the ‘gold standard’, across six different in silico variant scoring methods, different ethnic groups, and anatomical main groups of drugs. Prediction accuracies were well-validated and we found ethnicity as one of the essential parameters that improves the accuracy. Our GVB scoring method, which is a computational approach for integrating personal genomes with pharmacogenomics knowledge, may complement the current population-based approach, and vice versa.

## 4. Materials and Methods

### 4.1. Data Sets

We downloaded 2504 individual genomes from the 1000 Genomes Project, which comprises 26 ethnic subgroups [11]. No phenotype information was available for the 2504 genomes. Data about drugs and their targets, metabolic enzymes, transporters, and carriers were collected from the DrugBank [35], and then we selected 5099 drugs that had at least one PK/PD gene information. Drugs (5099), 3668 drug-related genes, and 19,038 drug-gene relations were included in the present study (Table 1).

### 4.2. Calculation of Gene-Wise Variant Burden (GVB) Scores for Genes and Drugs

Gene-wise deleterious coding variant burden (GVB) was calculated as described in our previous studies [5,6]. Several previous studies [11,12,13,14,15,36,37] have demonstrated that the impact of nonsynonymous coding variants on protein structure/function can be reliably predicted by applying empirical rules to the sequence, phylogenetic, and structural information characterizing the substitutions. An individual who carries a gene affected by deleterious coding variants may show an altered response to a drug with the PK/PD relationship with that gene. The deleterious variant scores of SIFT are used to define and derive the GVB scores. For each nonsynonymous coding variant, we computed the variant deleterious score of variant *i*, *S_vi_*, using the SIFT algorithm as follows:

(1) $$S_{v_{i}}=SIFT\left(v_{i}\right),\;if\;SIFT\left(v_{i}\right)\leq 0.7$$

where $SIFT\left(v_{i}\right)$ is an estimate of the deleterious effects of a variant $v_{i}$ on the gene/protein structure/function obtained using the SIFT algorithm.

Multiple nonsynonymous variants of the same gene may synergistically impact the structure/function of the gene. Gene-level *GVB*(*Gj*), the cumulative genic effect of all coding variants of the gene j, is defined as the geometric mean of the variant scores of all nonsynonymous variants in the coding region of the gene as follows:

(2) $$GVB\left(G_{j}\right)=\{\begin{array}{cc} 1 & ,if\;\left|G_{j}\right|=0\\ {\left(\underset{v_{i}\in G_{j}}{\prod }S_{v_{i}}\right)}^{\frac{1}{\left|G_{j}\right|}} & ,\;if\;\left|G_{j}\right|>0 \end{array}$$

where $G_{j}$ represents the set of all nonsynonymous coding variants of gene *j*.

For drug-level GVB of drug *k* *GVB*(*D_k_*), the cumulative effect of all genes related with a drug *k* is defined as the geometric mean of the GVB scores of all PK and PD genes of the drug extracted from the DrugBank database as follows:

(3) $$GVB\left(D_{k}\right)={\left(\underset{g_{j}\in D_{k}}{\prod }GVB\left(G_{j}\right)\right)}^{\frac{1}{\left|D_{k}\right|}}$$

where *D_k_* is the set of genes that interact with drug *k*. The SIFT score ranges from 0 to 1, with a lower score representing a more severe deleterious variant. Like variant scores, GVB scores range from 0 to 1, with lower scores representing more severe deleterious genes or altered drug responses.

### 4.3. Validation Using Known VDAs in PharmGKB

Direct GVB validation using the 2504 individuals of the 1000 Genomes Project is infeasible due to the lack of phenotype information or traceability. However, the existing pharmacogenomic knowledge base PharmGKB [9] can be used as the ‘gold standard’ for validation. We were able to identify 2364 PharmGKB associations that had at least one link to the 5099 study drugs. PharmGKB contains information on drug associations with both coding and noncoding variants. To avoid overlaps, we excluded 353 PharmGKB VDAs in the coding regions and used only 2011 VDAs in the noncoding regions to evaluate GVB obtained from coding variants. PharmGKB provides ethnicity information for some of its VDAs using the Office of Management and Budget (OMB) ethnicity classification scheme. We identified the ethnicity-specific VDAs in 1232 (61.3%) of the 2011 associations with at least one link to 246 (4.96%) of the 5099 drugs: 27 African-American, 86 Asian, 138 Caucasian, and the remaining 191 were unspecified.

We created a list of drug-level GVB ranks for the 5099 drugs from the DrugBank for each of the 2504 individuals of the 1000 Genome Project. Then, for each individual, we created a list of PharmGKB drugs that affected that person by mapping their sequence variants to the known pharmacogenomic variant-drug associations (VDAs) from PharmGKB.

The GVB score sensitivity and specificity were determined by comparing the GVB score ranks against the PharmGKB drugs from the known VDAs as the ‘gold standard’ for each individual at all score rank thresholds. This approach involves assigning the drug (i) a true-positive (negative) value if it is ranked above (below) a rank threshold and (not) found in the set of PharmGKB drugs and (ii) a false-positive (negative) if ranked higher (lower) than a threshold, but not (is) found in the set of PharmGKB drugs.

By counting the numbers of true- and false-positives and negatives at all the threshold cutoffs (or drug ranks) *L* for each of the 2504 individuals, we computed the drug rank sensitivity and specificity as follows:

(4) $$sensitivity=\frac{\left|D_{L}\cap GS\right|}{\left|GS\right|},specificity=1-\frac{\left|D_{L}-GS\right|}{\left|D-GS\right|}$$

where *D* represents the 5099 drugs, *GS* is the set of PharmGKB drugs used as the gold standard, and *D_L_* the set of drugs that appear above the threshold *L*. We also computed the AUCs for the 26 ethnic subgroups. It should be noted that we defined *GS* in both the ethnicity-specific and the non-specific settings. The ethnicity-specific *GS* was extracted from the VDAs reported in the same ethnic group as the individual, while the non-specific *GS,* from all of the VDAs regardless of ethnicity. The specificity, sensitivity, and area under the receiver operating characteristics curves were obtained using the R language ROCR package [38], R version 3.5.1.

### 4.4. Evaluation of GVB Derived from Other in Silico Variant Scoring Methods

For a comprehensive evaluation, we also computed GVB of the 5099 drugs for the 2504 subjects using five other in silico variant scoring methods (i.e., MutationTaster [14], PolyPhen-2 HDIV [15], PolyPhen-2 HVAR [15], phyloP [16], and GERP^++^ [17]). Several other scoring algorithms were excluded for evaluation by reason of right-skewed distribution (MutationAssessor [39], FATHMM [40] and Siphy [41]) or binary weighting scheme (LRT [42]). To normalize the GVB derived from the different scoring methods specified above, we transformed the score ranges from zero to one, with a lower score representing a more severe deleterious variant. After normalizing the GVBs, we performed the same validation steps on the long-known VDAs in the PharmGKB as mentioned above.

## Figures and Tables

**Table 1:** Number of drugs and gene-drug relations used in this study.

Drugs		Count
Total drugs		5099
Relations	Enzyme-drug relations	3477
	Transporter-drug relations	1772
	Carrier-drug relations	318
	Target-drug relations	13,471
	Total gene-drug relations	19,038

## Supplementary Materials

Can be found at https://www.mdpi.com/1422-0067/21/9/3091/s1. Table S1. ATC drug classes by anatomical main groups. Figure S1. Variant score distributions of the 2504 individual genomes of the 1000 Genome Project using six variant scoring algorithms. Figure S2. GVB distributions of genes and drugs of the 2504 individual genomes of the 1000 Genome Project. using six variant scoring algorithms. GVB distributions of (A) 3668 drug-related genes and (B) 5099 drugs. Figure S3. Evaluation of different anatomical main groups using the Mutation Taster algorithm. Figure S4. Evaluation of different anatomical main groups using the Polyphen-2 HVAR algorithm. Figure S5. Evaluation of different anatomical main groups using the Polyphen-2 HDIV algorithm. Figure S6. Evaluation of different anatomical main groups using the PhyloP algorithm. Figure S7. Evaluation of different anatomical main groups using the GERP^++^ algorithm.

## Abbreviations

ADR	Adverse drug reaction
GWAS	Genome-wide association study
VDAs	Variant-drug associations
NGS	Next generation sequencing
GVB	Gene-wise variant burden
AUC	Area under the receiver operating characteristics curve
PK	Pharmacokinetics
PD	Pharmacodynamics
ROC	Receiver operating characteristic
TP	True-positive
TN	True-negative
FP	False-positive
FN	False-negative
GS	Gold standard
5′-UTR	5′-untranslated region
3′-UTR	3′-untranslated region
VKORC	Vitamin K epoxide reductase complex
BAR2	Beta2-adrenergic receptor
OMB	Office of management and budget
