# Supplementary material for: Gene-Wise Burden of Coding Variants Correlates to Noncoding Pharmacogenetic Risk Variants
Source: Int J Mol Sci. 2020 Apr 27;21(9):3091. doi: 10.3390/ijms21093091 (PMC7247590; doi:10.3390/ijms21093091)
Supplement: Supplementary file 1 [file ijms-21-03091-s001.zip › GVB_Drug_Supplement/TableS1.docx]

| Drug class | ATC code | No. of drugs | No. of VDAs in PharmGKB | No. of Drugs in PharmGKB |
| --- | --- | --- | --- | --- |
| Alimentary tract and metabolism | A | 151 | 126 | 21 |
| Blood and blood-forming organs | B | 66 | 48 | 8 |
| Cardiovascular system | C | 191 | 258 | 43 |
| Dermatologicals | D | 105 | 60 | 10 |
| Genitourinary system and sex hormones | G | 82 | 0 | 0 |
| Systemic hormonal preparations, excl. sex hormones and insulin | H | 38 | 0 | 0 |
| Anti-infectives for systemic use | J | 148 | 108 | 18 |
| Antineoplastic and immunomodulating agents | L | 180 | 384 | 64 |
| Musculoskeletal system | M | 63 | 72 | 12 |
| Nervous system | N | 271 | 456 | 76 |
| Antiparasitic products, insecticides, and repellents | P | 30 | 30 | 5 |
| Respiratory system | R | 113 | 66 | 11 |
| Sensory organs | S | 115 | 54 | 9 |
| Various | V | 33 | 12 | 2 |

**Table S1. ATC drug classes by anatomical main groups.**
